# Adipose tissue stem cells in regenerative medicine

**DOI:** 10.3332/ecancer.2018.822

**Published:** 2018-03-28

**Authors:** Vanesa Verónica Miana, Elio A Prieto González

**Affiliations:** Centre for Advanced Studies in Humanities and Health Sciences, Interamerican Open University, Buenos Aires, Argentina

**Keywords:** adipose tissue stem cells, reconstructive surgery, secretome, stem cell treatment, cancer

## Abstract

Adipose tissue-derived stem cells (ADSCs) are mesenchymal cells with the capacity for self-renewal and multipotential differentiation. This multipotentiality allows them to become adipocytes, chondrocytes, myocytes, osteoblasts and neurocytes among other cell lineages. Stem cells and, in particular, adipose tissue-derived cells, play a key role in reconstructive or tissue engineering medicine as they have already proven effective in developing new treatments. The purpose of this work is to review the applications of ADSCs in various areas of regenerative medicine, as well as some of the risks associated with treatment with ADSCs in neoplastic disease.

## Introduction

Stem cells can be classified by their origin as: (a) embryonic; (b) fetal; (c) adult and (d) induced pluripotent. The classification can be simplified as embryonic and adult mesenchymal [1].

Human embryonic stem cells (ES cells) are derived from the inner layer of the blastocyst and, due to their pluripotency, are used in tissue engineering and regenerative medicine. Human fetal mesenchymal stem cells (hfMSCs) can be harvested from stem cells present in the amniotic fluid or the umbilical cord. They are multipotent, but like the embryonic cells they present difficulties in harvesting them due to their limited availability and also due to ethical issues. The induced pluripotent stem cells (iPSCs) do not present major limitations in terms of harvesting, and the greatest difficulty in terms of their clinical use is related to the induction procedures in the laboratory to differentiate them into specific cells needed for the treatment of certain diseases [2]. For this reason, adult stem cells are the most promising for use in clinical practice and in research on the basic aspects of this cellular compartment.

Adipose tissue-derived stem cells (ADSCs) are mesenchymal cells, which have a capacity for self-renewal and which can also be differentiated into adipocytes, chondrocytes, myocytes, osteoblasts and neurocytes among other cell lineages [3], which has resulted in them being used in clinical trials for the treatment of conditions such as diabetes mellitus, liver disease, corneal lesions, articular and cutaneous lesions, among others [4–10]. In addition, stem cells and, in particular, adipose tissue-derived cells, play a key role in reconstructive or tissue engineering medicine as they can be used to develop new treatments. [11].

Among the advantages of ADSCs, the greater ease of access and harvesting by means such as subcutaneous lipoaspiration, a much less painful procedure than harvesting bone marrow stem cells, and their use, is less associated with ethical controversies because they are harvested from autologous fat, unlike ES cells [12].

Adipose tissue has been one of the most studied tissues in the last decade due to its endocrine activity which is manifested in the release of adipocytokines, cytokines, transcriptional and growth factors, which forms a secretome [11, 13, 14]. Adipose tissue is no longer only considered an energy reservoir, thermal insulator or mechanical buffer, but its participation in a complex network of interactions with the endocrine, nervous and cardiovascular systems has been highlighted. It is a tissue that originates in the mesoderm and is formed by adipocytes and a fraction of stromal cells that include vascular smooth muscle cells, endothelial cells, fibroblasts, monocytes, macrophages, pre-adipocyte lymphocytes and ADSCs. ADSCs may undergo differentiation to mesodermal or trans-mesodermal lineages and give rise to cells that are naturally of ectodermal origin [1].

The procedure based on the separation of the vascular stroma contained in adipose tissue has allowed access to stem cells without resorting to embryonic tissue, facilitating its use in regenerative medicine [1, 12]. The (CD34 +) ADSCs are those that can differentiate and form nonhematopoietic colonies, in turn they form a compartment in which various subpopulations are identified: pro endothelial (CD146 + / CD31 + / CD34 +); pericytes (CD146 + / CD31– / CD34–), a transient subpopulation (CD146 + / CD31– / CD34 +); and stromal cells that show a greater potential to form adipocytes (CD146– / CD31– / CD34 +) [15].

In recent years, it has been demonstrated that by using specific inducers in the laboratory, these adipose-derived stem cells (ADSCs) have the ability to differentiate into the cellular line needed [12, 16].

The purpose of this project is to review the applications of adipose tissue stem cells in different conditions including cancer, as well as to highlight some problems associated with the use of these cells in oncology and especially in post-surgical tissue reconstruction.

## Methods

This review project was carried out through the evaluation of biomedical journals indexed in MEDLINE, PubMed, Scielo, SIIC and Redalic using search terms such as ‘tissue engineering and oncology’, ‘regenerative medicine’, ‘biomaterials’, ‘adipose tissue’ and ‘stem cells’.

The review included articles in English and Spanish and publications from the last 5 years, as well as other less recent ones that, due to their importance or uniqueness, seemed relevant to us.

## Adipose tissue stem cells in regenerative medicine

### Characteristics of stem cell harvesting

Adipose tissue is a specific variety of connective tissue; composed of a group of cells called adipocytes, specialised in storing fats. The adipose tissue consists of the joining, through reticular fibres, of the adipocytes forming lobes, between which run numerous blood vessels.

Adipose tissue adult stem cells, termed stem cells or mesenchymal stem cells (MSCs), can be harvested through surgery and direct excision, liposuction in the trunk and extremities as well as the Coleman technique for fat tissue transplantation and remodelling. This technique has shown a higher yield of viable adipocytes than conventional liposuction [1, 17, 18]. In liposuction, the unwanted fat of a part of the body is removed by aspiration and injected in the areas where increased volume is required or to achieve a cosmetic improvement. [19, 20]. The isolation of ADSCs is carried out from the lipoaspirated material where the cells of the stromal vascular fraction (SVF) can be found; from this fraction, different cell types can be isolated after washing, enzymatic digestion and centrifugation of the samples [16, 21, 22]. The cell types found in SVF include: preadipocytes, fibroblasts, adult mesenchymal stem cells, monocytes, macrophages, lymphocytes as well as pericytes related to angiogenesis. [23–25].

### Harvesting cell lines

Maintaining cultures of mesenchymal cell lines allows a greater quantity of pluripotent stem cells (PSC) to be harvested. However, at this time there is much controversy about the most efficient methods of cell derivation and reprogramming and which culture conditions allow them to maintain their undifferentiated state in an unlimited way. Cultures that could retain a stable phenotype could be induced to differentiate into a specific cell lineage/tissue in a specific and reproducible manner at the desired time [12, 26, 27].

The main challenge for the application of these cells in future cell replacement therapies is to be able to control their differentiation in specific tissues. In this sense, there are a multitude of cultivation methods, reprogramming strategies, genetic manipulation, epigenetic modulation as well as organisation in three-dimensional matrices and directing stem cells to the areas where they may be needed [12, 28, 29].

The feeder cells that have been most used in the cultivation of human PSCs are mouse embryonic fibroblasts (MEFs) [30]. These cells secrete nutrients into the medium and form a single layer, acting as a cellular support on which the PSCs are placed. Recently, the use of feeder layers of nonhuman origin has begun to be ruled out as well as the use of a medium conditioned by feeder cells due to the suspicion that they can be carriers of murine viruses [31].

The feeder layers that have replaced them are made up of human embryonic fibroblasts and the extra cellular medium that these have conditioned. There is a remarkable input of cytokines, growth factors among others, that enrich or condition the cultivation medium with elements from the same species. The co-cultivation of stem cells and human feeder cells is safer and is a model which is closer to natural conditions. Another approach to the problem of guaranteeing the suitability of the cultures is the development of serum-free and xenobiotic-free media [31].

Tissue regeneration using stem cells has incorporated three-dimensional matrices constructed by different biomaterials to provide a microenvironment closer to that of the natural histoarchitecture. Matrices along with growth factors, such as bone morphogenetic protein 2 and culture expanded adipose stem cells, are used in tissue reconstruction, for example, after cancer surgery [32].

Biomaterials are important in the culture of SCs as they are three-dimensional polymeric structures used to achieve an organisation of cell growth, closer to that of tissues [33–35]. Among the materials used to assemble the three-dimensional biomatrices so that the stem cells can embed, we can include silica, collagen and hyaluronic acid that interacts with the SCs through integrin-like proteins. The biomatrices formed by collagen increase the range of differentiation possibilities in different cell lineages such as cartilage, bone, skin and lung, while others formed by polysaccharides that are not found in the extracellular matrix limit their possibilities of differentiation [34]. The size of the particles or the density of the networks that are developed by various biomaterials such as silica that influences the growth and differentiation potential of ADSCs. It has been proven that kinase systems are involved in transducing signals generated by cell contact with the biomatrices. The mitogen-activated kinases seem to be the base of the proliferative effects of such culture conditions and may be related to the acquisition of malignant phenotype of ADSCs in experimental models or in reconstructive surgery [36], as we will see later.

### Cultivation and genomic alterations of cultured stem cells

The appearance of mutations in genes controlling the integrity of DNA promotes genomic instability, which leads to diversification into cellular subpopulations that in turn can accumulate further mutations and give rise to cell genotypes that can express the tumour phenotype [37].

The cells transformed through cultivation mimic the tumour phenotype *in vivo*. It is then necessary to evaluate the genetic stability of cultures that have therapeutic purposes.

Maitra *et al* [38] studied genomic stability in human ESM cell lines and compared the frequency of mutations between the early and late passages (after the eighth or ninth passage). In late passages, an increase in the number of genomic alterations was observed that coincided with some of those that appear in malignant tumours, including aneuploidies, mutations in mitochondrial DNA and alterations in methylation. The fact that stem cell lines develop genetic and epigenetic alterations *in vitro* implies the need to use them before derivations that could increase their oncogenic potential produced in cultivation [37].

According to Gimble et al [39], five criteria must be followed for the use of these cells for medical purposes: (a) Presence of cells in abundance. (b) Harvesting through minimally invasive procedures. (c) Regulation of the differentiation of cell lineages. (d) Possibility of their use as autograft. (e) They must be manipulable within the rules of professional practice.

### ADSCs in experimental and human studies

Handling in the laboratory and their transfer and inoculation in the operating room must be done in compliance with biosafety regulations and respecting regulations concerning the ethics of therapeutic procedures.

The animal model test records are abundant, for that reason some are selected, by way of illustration, in a growing field where one cannot claim to be exhaustive.

ADSCs have been used in experiments in a model of osteoporosis in rats in which differentiated stem cells were implanted to regenerate bone tissue. Over a period of 2 years, regeneration of affected bone tissue was achieved [40–42, 45–47].

Bacou *et al* [43] documented a study on rats in which cells differentiated from ADSCs were injected into the injured tibialis anterior muscle in order to evaluate whether muscle tissue regeneration occurred; after 60 days in the treated group, the cross-section of muscle and maximal contraction force increased compared to the untreated control group. Meanwhile, another author reported the production of dystrophin in mice, in a model of Duchenne muscular dystrophy, when ADSCs were transplanted [44].

In a model of autoimmune thyroiditis in C57B/6 mice, the therapeutic effects of ADSC transplants were studied from mice of the same line (syngenic) and BALB/c (allogenic) mice. In both cases, with both allogeneic and syngeneic ADSCs, there was a decrease in the amount of antithyroglobulin autoantibodies as well as inflammatory response and the balance between Th1/Th2 was restored [48].

In a model of rheumatoid arthritis in rats, the syngenic and allogenic transplantation of mouse or human ADSCs resulted in less cartilage damage and decreased antibodies against mouse collagen II, as well as interleukin 6 in the treated groups [49]. Research on murine models has been published in which repopulation of the pancreas was observed with cell aggregates similar to the islets of Langerhans, which were formed from ADSCs and are capable of secreting insulin [50, 51].

### In vivo and clinical trials in the cardiac muscle

Attempts were made to repopulate areas of the cardiac muscle after a heart attack in both animal models and humans. Tests were conducted by injecting stem cells directly into the site of the post-infarction lesion with the purpose of restoring the thickness and elasticity of the walls, alterations that often result in ventricular failure. However, the results have not been satisfactory. Apparently, the absence of a syncytium in which the transplanted cells can be fixed and proliferate is one of the causes of these failures, and so several biomatrices have been introduced, such as those based on peptide nanofibers capable of self-assembly in three-dimensional networks and with better results in retention and proliferation of the transplanted cells [52].

ADSCs have greater capacity for differentiation into epithelial cells and promotion of angiogenesis; it is known that hypoxia favours the secretion of growth factors that contribute to the proliferation of myocardiocytes and the reduction of infarcted areas. It has also been observed that fat cells originating from epicardial fat are phenotypically closer to myocardiocytes and consequently easier to differentiate in such cardiac cells than in those harvested in other regions, such as omental fat. The paracrine activity of these cells’ secretome also favours differentiation into myocardiocytes [53].

Other authors report that ADSCs permeabilised and treated with factors secreted by myocardiocytes have increased their proliferative capacity at the transplant site and secreted cardiac cell markers, such as troponin, sarcomeric alpha actinin and desmin [54, 59, 82].

The genetic manipulation of ADSCs is a pathway that is being explored in experimental models involving research and treatment of different liver or heart conditions [55–58]. An example of this approach in which transgenesis and ADSC therapy cross is the study in which they have been transduced with the haem oxygenase 1 gene, the product of which decreased oxidative damage and apoptosis of myocardiocytes. In a model of myocardial infarction in rabbits, modified ADSCs were transplanted in order to over-express the enzyme while a control group was treated with unmodified cells. When they were evaluated 4 weeks after the treatment, those that had received modified cells expressed a greater quantity of markers such as connexin 43 and tyrosine hydroxylase as well as greater differentiation towards myocardiocytes, increase in angiogenesis, a decrease in post-infarction scars and a growth of sympathetic nerves [59].

### Clinical trials in bone tissue

In patients with bone deficiencies in the jaws, who were treated with ADSCs in addition to a bone matrix and autologous fibrin, when the grafts were checked in a tomography performed 90 days after the procedure, bone growth was checked in the deficit areas [60, 61].

An example of the application of ADSC transplants in reconstructive medicine has been the restorative treatment of the cranial vault of a 7-year-old girl after a serious injury, with bone resorption in which a decompressive craniotomy had to be performed, leaving a cranial defect of 120 cm^2^ as a sequela. The patient was treated with 15 ml iliac cancellous bone supplemented with 10 ml of ADSCs that were harvested and isolated during the surgical procedure. A tissue adhesive consisting of autologous fibrin glue and resorbable mesh was used to increase graft stability. Monitoring with posterior tomography showed a significant reossification at 3 months which made it possible to remove the protection that had been inserted the previous year at the time of the injury. [61]

ADSCs have the ability to differentiate into the cell line that is needed for the regenerative treatment of different conditions such as coronary disease, osteoporosis, bone regeneration of the jaw and the vestibular table, in amyotrophic lateral sclerosis, osteogenesis imperfecta, Crohn’s disease (closure of entire cutaneous fistulas), alopecia, graft versus host disease and diabetes mellitus [62–64].

This means that the field of experimental research on the application of ADSCs is expanding and parallel clinical trials are developing, which leads to an experimental and clinical complementation bringing cell therapy closer to the standards of mainstream medical practice. On this website, clinical trials with ADSCs that are currently in progress may be consulted (https://clinicaltrials.gov/adipose%20derived%20stem%20cells).

### ADSCs in cancer therapy

In recent years, concerning the use of these cells for the treatment of different cancers, evidence indicates that these cells may have a pro-tumourigenic or anti-tumourigenic role, depending on different factors such as the types of ADSCs, their origin, the cell line of the cancer studied, the interactions between ADSCs and the cells of the host immune system [65].

In one way or another, the effects are related to a set of biological actions of the SC whose determinants are not yet well understood. SCs can promote the expression of growth factors as well as the development of tumour stem cells. The pro-tumourigenicity of the SC is explained by the formation of blood vessels;, and also, by the increased survival of tumour stem cells, by decreasing body’s immune response to the tumour and contributing to the formation of metastases. The origin of SCs is a factor that conditions the response to different types of tumours, which further complicates the picture [66–68].

Regarding antitumour actions, there are reports in experimental models that include melanomas, pancreatic cancer and prostate cancer about which stem cells of different origins, such as bone marrow and adipose tissue, were able to cause tumour size reduction by mechanisms that included the inhibition of angiogenesis, apoptosis and interference with cell proliferation, which is the mechanism used to explain the effect on prostate cancer in nude mice treated with ADSCs [65].

Other approaches to use ADSCs in Oncology include the encapsulation of ADSCs to protect them and guarantee that they can serve as vectors for antineoplastic drugs taking advantage of the tropism of migration towards the primary tumour or its metastasis [68]. The use of genetically-modified SCs to carry tumouricidal genes can serve as a ‘magic bullet’ against neoplastic cells. In experimental murine models, positive results have been obtained in gliomas, Kaposi’s sarcoma, melanoma or lung cancer. Interleukins and interferons are among the factors that can be carried by SCs to the tumour site. It is also worth mentioning that the suicide gene therapy is based on sending inactive prodrugs together with cells modified to express transgenic enzymes that can modify the precursors, making them toxic in the tumour site [69].

### ADSC therapy and oncogenic risk

Within the tumour structure are the parenchyma and the stroma involving the vessels and the connective tissue. The stroma is of great importance in the nutrition of tumour cells [68, 70]. The stroma participates in the regulation of growth and spread of a tumour through interaction with parenchymal cells. Among the cells associated with the tumour are myofibroblasts that have a role in metastasis by stimulating angiogenesis via the stromal factor-1 (SDF-1) and secretion of metalloproteases that help to reshape the extracellular matrix [71, 72].

A potential source of stromal cells is multipotent mesenchymal stem cells [25, 73, 74]. In addition to their recognised characteristics of multipotentiality and self-renewal, stem cells show tropism of migration towards tumour cells, which is an advantage for treatment although at the same time it increases the possibility of recruitment towards the tumour phenotype as a consequence of paracrine stimuli [75].

The mechanisms that can explain the role of MSCs in tumour progression include the decrease in the immunological reactivity of tumour cells which leads to a greater spread of cancer. The molecules secreted by the neoplastic cells are the carriers of the communication that results in the modification of the behaviour of the stromal cells, their recruitment and further modulation of the tumour phenotype [76]. In addition to the secretion pathways that characterise paracrine stimulation, there are exosomes that are nanoscale vessels originating in intracellular multivesicular bodies and that have been found in reticulocytes, enterocytes, tumour cells and SCs among others. These vessels contain proteins that are related to the presentation of antigens of the major histocompatibility complex, as well as to signal transduction, migration and cell adhesion. They also contain heat shock proteins and MFG-E8 or lactaderin, which is related to the phagocytosis of apoptotic cells. It is striking that they include mRNA and microRNA that are functional in the target cells [77–79].

Exosomes are a paracrine pathway that involves the massive transport of proteins and the induction of pleiotropic cellular responses, participate in the crosstalk between SCs and other cell types and function in pro-tumourigenic recruitment and activation of remaining cancer cells, after cancer therapy, can exert on tissues carrying mesenchymal stem cells. This becomes important when an autograft or allograft of adipose tissue is performed in breast reconstruction following cancer surgery [11, 80].

In cancer patients with a recommendation for breast reconstruction, the surgical option with transverse rectus abdominis muscle (TRAM) flap is one of the most used for breast reconstruction after performing a radical mastectomy. The use of stem cells in the reconstruction process reduces the appearance of fat necrosis around the flap. Stem cells improve the viability of the TRAM flap and significantly reduce peripheral tissue necrosis [81, 82].

Despite fears about the risks of malignant transformation that may be associated with the use of autologous ADSC, very low frequencies of loco-regional tumour recurrences have been reported in post-surgery breast cancer reconstruction. [83, 84] This contradicts the experimental results in which the oncogenic potential of the ADSC secretoma is demonstrated, and also, expresses the great complexity and heterogeneity of the factors that can promote or antagonise the malignant transformation when ADSC is transplanted [85, 86].

### Effect of ADSC on scarring

There are several stages of scarring that, from the biochemical point of view, are characterised by the release of different growth factors. In the acute phase, transforming growth factor beta (TGFβ), granulocyte colony stimulating factor, platelet derived growth factor (PDGF) and inflammatory cytokines are identified. In the cell proliferation phase in which the granulation tissue is formed, the epidermal growth factor (EGF), the tumour necrosis factor, the vascular endothelial growth factor, the growth of keratinocytes (KGF), insulin growth factor, nervous growth factor, as well as inflammatory interleukins. In the final or remodelling stage, factors that induce epithelialisation are produced, such as KGF, TGFβ, PDGF, EGF and hepatocyte growth factor [87, 88].

It is worth noting that a large part of the therapeutic effect of ADSC can be explained by the endocrine activity of adipose tissue. An example of which is the fact that, by decreasing blood flow, as in the case of flap reconstruction and hypoxia, ADSCs increase the production of fibroblast growth factor while increasing the secretion of angiogenic factors that contribute to revascularisation. Evidence abounds that the factors secreted by stem cells have therapeutic effects that are expressed in tissue regeneration and are also effective in other aspects, such as in the reduction in neuropathic pain in diabetes [86–89, 90–93].

In a model of excisional scars in BALB/c mice, the factors secreted by ADSCs were able to increase the speed of healing and attenuate the deposit of collagen. In the same study, it is reported that the incubation of fibroblasts and tissue from hypertrophic scars, with a medium conditioned by ADSCs, decreased the expression of alpha actin and of collagens I and II; whose fibres were finer and more organised than those of untreated cultures. The meaning of these results, the reduction of cutaneous fibrosis that generates deforming scars, cannot be minimised [94].

### Other research avenues involving stem cell therapy

The information reviewed here allows us to affirm that there is an increase in the possibilities of ADSC use in conditions in which, for different reasons, there are injuries with total or partial tissue loss. In this sense, the projections include traumas with avulsion wounds, fractures in which bone mass is destroyed and burns, which areamong others [95, 96].

In some neurodegenerative diseases, the capacity of genetically modified ADSC is explored in order to counteract the cytotoxic effects of substances generated in patient’s cells [97].

In autoimmune conditions, the ability to interfere with the alterations of humoral and cellular immunity provides an avenue of research that is already gathering some promising results in the medium term [98, 99].

Research into the possibilities of stem cells intersects with other areas such as transgenesis and gene editing. In this sense, not only it is intended to induce the differentiation of stem cells in specific lineages according to therapeutic needs but also stem cells can be modified at the genetic level, so that cell therapy also becomes a gene therapy which can be used to correct defects such as congenital metabolism errors resulting in enzymatic deficits or structural proteins. Stem cells could function as vectors for the transduction of corrective genes [100–102]. The epigenetic modulation of SCs before and after they have been introduced into the recipient is another avenue that should be explored, especially in those conditions in which errors that require regulation of the activity of the transplanted cells must be corrected [103, 104].

Regenerative medicine and, within it, reconstructive surgery where cellular and molecular biology intersect, with the proper surgical restorative procedures, will contribute to shape the medicine of the near future. Although not ready in the near future, the restructuring or regeneration of parts of organs or whole organs has a solid base in recent stem cell therapy results.

## Conclusions

ADSCs are an accessible and more flexible alternative for treating conditions that require tissue regeneration. Their therapeutic efficacy is founded on the applications based on these cells not only in experimental models in animals but also in an increasing number of human trials.

Conditions that can be treated with ADSCs range from traumatic injuries, through to neurodegenerative and endocrine metabolic disorders, and postsurgical reconstructions.

In relation to antineoplastic treatment, the paracrine and endocrine stimuli mediated by the secretomas of the ADSC may be beneficial, but may also promote tumour progression.

The development of the use of ADSCs in the treatment of highly prevalent conditions involves not only cell transplantation, but intersects with genetic manipulation, epigenetic modulation and the effect of secretomas correcting pathophysiological alterations.
